# Intratumoral neutrophil extracellular traps are associated with unfavorable clinical outcomes and immunogenic context in pancreatic ductal adenocarcinoma

**DOI:** 10.3389/fimmu.2022.1027459

**Published:** 2022-10-17

**Authors:** Xianlong Chen, Heng Ma, Shengwei Mo, Shuangni Yu, Zhaohui Lu, Jie Chen

**Affiliations:** Department of Pathology, Peking Union Medical College Hospital, Chinese Academy of Medical Sciences & Peking Union Medical College, Beijing, China

**Keywords:** adjuvant chemotherapy, extracellular trap, immune checkpoint, macrophage, neutrophil, pancreatic ductal adenocarcinoma, prognosis

## Abstract

Extracellular traps (ETs) and tumor-infiltrating immune cells play crucial roles in tumor progression. However, little is known about the clinical significance of tumor-infiltrating neutrophils and macrophages and the related ETs in pancreatic ductal adenocarcinoma (PDAC). This study investigates the associations between neutrophil or macrophage infiltration or ET formation and the clinicopathological features, molecular characteristics, immune checkpoint molecules, clinical outcomes, and response to adjuvant chemotherapy (ACT) in PDAC. We performed multiplex immunofluorescence staining to detect ET formation by neutrophils or macrophages using tissue microarrays obtained from 205 patients, and analyzed the immunohistochemistry data for PD-L1, PD-L2, B7-H3, and B7-H4. The ET expression rates in macrophages and neutrophils were 23.9% and 45.4%, respectively. Patients with a high density of neutrophils or positive expression of neutrophil ETs exhibited poorer progression-free survival (PFS) and disease-specific survival (DSS), whereas macrophage ETs were not related to PFS and DSS. Neutrophil infiltration and ET formation were identified as independent prognostic predictors of DSS using univariate and multivariate Cox analyses. Patients with PDAC with lower neutrophil infiltration or negative staining for neutrophil ETs are more likely to benefit from ACT. Patients with PDAC were more accurately stratified based on the infiltration of neutrophils and presence of neutrophil ETs, and patients with low neutrophil infiltration and negative staining for neutrophil ETs showed the best survival. Patients with positive neutrophil ETs demonstrated inferior DSS compared to those with negative neutrophil ETs in the PD-L1 tumor proportion score (TPS) < 1% and PD-L1 IC < 1% subgroups. However, the positive expression of neutrophil ETs was not related to DSS in the PD-L1 TPS ≥ 1% or PD-L1 IC ≥ 1% subgroup. Our findings emphasize the potential of neutrophil infiltration and ETs as prognostic markers that could guide the formulation of more effective personalized treatments for PDAC.

## Introduction

Pancreatic ductal adenocarcinoma (PDAC) is the most common malignant tumor of the pancreas with a 5-year survival rate of less than 8% (1, 2). For the ~20% of PDAC patients that are eligible for surgery (3), the 5-year survival rate is lower than 20%, and 80% of patients experience recurrence within two years (2, 3). Although adjuvant chemotherapy (ACT) is the standard treatment for resectable PDAC (3), the response to treatment varies among patients because of inter- and intra-tumoral heterogeneity (3). Therefore, an urgent need exists for reliable predictive tools that allow for personalized treatment based on a patient’s condition.

The tumor microenvironment of PDAC, comprising tumor cells, immune cells, endothelial cells, fibroblasts, and extracellular substances, plays a vital role in tumor progression and presents several therapeutic targets (4, 5). The importance of immune cells, such as neutrophils and macrophages, in tumor progression and resistance to immunotherapy is well established (6). Tumor-infiltrating macrophages contribute to angiogenesis, tumor invasion, metastasis, and resistance to chemotherapy (7, 8); tumor-infiltrating neutrophils participate in immunosuppression, modification of the extracellular matrix, and tumor progression (8).

Immune cell-related extracellular traps (ETs) also influence disease progression and have been used as indicators of prognosis and therapeutic targets in various diseases (9). ET formation is a form of cell death and is characterized by the production of extracellular webs, consisting of nuclear DNA and granular and cytoplasmic proteins by immune cells after infection, surgery, radiation, or chemotherapy (10). Neutrophils and macrophages are known to produce ETs (11, 12). Neutrophil ETs are involved in many tumor processes (9), they promote tumor cell growth in hepatocellular carcinoma (13), and promote metastasis (14). Neutrophil ETs are also involved in mediating the suppression of anti-tumor immune cells. In colorectal adenocarcinoma, neutrophil ETs promoted T cell exhaustion in the tumor microenvironment (15), and their inhibition improved anti-PD-1 immunotherapy through the PDL-1/PD-1 axis by increasing the density and cytotoxicity of CD8 T cells (16). Neutrophil ETs contribute to fostering tumor spread at distant sites and tumor initiation, growth, progression, and angiogenesis in some types of cancer (17). Beyond their roles in cancer, Neutrophil ETs also play vital roles in different infectious diseases, including Cryptococcus neoformans infection, Chagas disease, severe acute respiratory syndrome coronavirus 2, leprosy type 2 reactions, and some virus infection (18–22). Macrophage ETs are involved in acute kidney injury, pathological conditions characterized by excessive hypochlorous acid formation, and antibacterial immunity (11, 23). In PDAC, the clinical significance of neutrophil and macrophage ETs and their association with immune checkpoint molecules and immune cell infiltration remains unclear.

This study investigates the associations of infiltration or ET formation by neutrophils or macrophages with the expression of immune checkpoint molecules (PD-L1, PD-L2, B7-H3, and B7-H4), clinicopathological features, clinical outcomes, and response to ACT in PDAC. We used multiplex immunofluorescence, digital imaging techniques, and immunohistochemistry to detect the levels and distribution of neutrophil and macrophage infiltration and ETs in PDAC tissues.

## Materials and methods

### Patients and tissue microarrays

A total of 205 patients with PDAC who underwent standard surgical procedures, including classic pancreaticoduodenectomy, pylorus-preserving pancreaticoduodenectomy, distal pancreatectomy, or total pancreatectomy between 2015 and 2019 at the Peking Union Medical College Hospital (PUMCH, Beijing, China) were enrolled in this retrospective cohort study. Tumor differentiation, perineural invasion, lymphovascular invasion, and lymph node metastasis were determined from histopathological slides by two expert pathologists (SNY and ZHL), according to the 5th edition of the World Health Organization Classification of Tumors of the Digestive System. The stage for each patient was determined on the basis of the American Joint Committee on Cancer (AJCC) 8th edition. Patients with ACT were defined as patients who received at least one cycle of 5-fluorouracil and/or gemcitabine-based ACT. Other clinical data were collected from the medical records. Survival information was obtained from the medical records and telephone interviews. The time between surgery and tumor progression or the last follow-up appointment (October 10, 2020) was defined as the progression-free survival (PFS) rate. Disease-specific survival (DSS) was calculated from the date of surgery to the time of death or last follow-up. This study was approved by the Institutional Review Board of PUMCH (S-K1593) and was conducted in accordance with the Declaration of Helsinki. Informed consent was obtained from all patients.

After reviewing hematoxylin–eosin-stained slides, TMA cores with a diameter of 2 mm were obtained from the corresponding formalin-fixed paraffin-embedded blocks using a Tissue Microarrayer (MiniCore, Mitogen, Hertford, UK). All tumor spots were punched from the center of the specimen.

### Multiplex immunofluorescence staining

Using an Opal 7-color Kit (Akoya Biosciences, Marlborough, MA, USA), multiplex immunofluorescence staining was conducted on TMA sections according to the manufacturer’s protocol and as previously described by Yeong et al. (24) for simultaneous detection of CD68, myeloperoxidase (MPO), citrullinated histone H3, and DAPI. The TMA sections were baked at 65°C for 2 h and subjected to deparaffinization, rehydration, and heat-induced epitope retrieval. The sections were then incubated with a primary antibody for 1 h at room temperature, followed by incubation with an anti-rabbit horseradish peroxidase (HRP)-conjugated secondary antibody (Akoya Biosciences). We incubated the slides with an opal fluorophore-conjugated tyramide signal amplification reagent (Akoya Biosciences). Heat-induced epitope retrieval was performed to remove the bound antibody complexes. The same procedure was repeated until all targets were detected, and the samples were labeled with DAPI (Akoya Biosciences). The following antibodies were used: anti-CD68 (D4B9C; Cell Signaling Technology, Danvers, MA, USA)/Opal 520, anti-citrullinated histone H3/Opal 620 (ab5103; Abcam, Cambridge, UK), MPO (E1E7I; Cell Signaling Technology, Danvers, MA, USA)/Opal 690. Finally, the sections were mounted using a hard-set medium.

### Multispectral imaging and scoring

The multiplex-stained TMA slides were scanned using a Vectra Polaris system (Akoya Biosciences). Spectral unmixing and multispectral tissue imaging were performed using InForm software (Akoya Biosciences). Based on the TMA mode, the entire field of view for every core in each TMA section was used for image analysis. Cell segmentation was performed according to nuclear detection using DAPI staining. All scoring was independently performed by two investigators (XL. C. and H. M.) who were blinded to the patients’ clinicopathological data. Positive MPO ETs were identified by co-staining with MPO and citrullinated histone H3, and macrophage ETs were identified by co-staining with CD68 and citrullinated histone H3 (25). Given that MPO and citrullinated histone H3 are also present in macrophage ETs, neutrophil ETs were calculated by subtracting macrophage ETs from MPO(+) ETs (12, 26). Four random fields of view for ETs in each section were randomly acquired at 200 × multispectral images for quantitative digital analysis. The mean counts of four fields were used for statistical analysis. We identified positive neutrophil ETs when neutrophil ETs were ≥ 8. And we identified positive macrophage ETs when macrophage ETs were ≥ 10. The cut-off points of 8 and 10 were set using X-tile (Yale University, New Haven, CT, USA) and were the best values for prognosis discrimination determined through preliminary analysis of our cohort. X-tile is a bio-informatics software for biomarker assessment and outcome-based cut-point optimization.

### Immunohistochemistry and evaluation

The following primary antibodies were used: CD15 (SP159; Abcam), CD68 (D4B9C; CST), B7-H3 (D9M2L, CST), B7-H4 (D1M8I, CST), PD-L2 (18251-1-AP; Proteintech, Chicago, IL, USA)All slides were automatically stained using a BOND-III immunostaining instrument (Leica Biosystems, Wetzlar, Germany) according to the manufacturer’s instructions.

Immunostaining was assessed by two trained pathologists who were blinded to the patients’ clinicopathological information and clinical outcomes. In cases of discrepancy, a third gastroenteropancreatic subspecialty pathologist made the final decision. Staining of PD-L1 was evaluated according to the percentage of positives tumor cells (tumor proportion score, TPS) or immune cells (IC value). Infiltrating mononuclear cells in the tumor microenvironment were considered as immune cells. Samples were segregated into two groups: < 1% or ≥ 1% of positive cells (27–30). To evaluate PD-L2, B7-H3, and B7-H4 staining on tumor cells, X-tile software set the cutoff of 1% as the best value for prognosis discrimination determined through preliminary analysis of our cohort. The density of neutrophils and macrophages in the stroma was quantified at 400× magnification using a computerized imaging system (Hamamatsu Photonics, Hamamatsu City, Japan). Four fields of maximal concentration of neutrophils and macrophages at ×400 magnification were selected using digitally scanned images. The mean counts of 4 fields were used for quantitative digital analysis. We used medians as cut-off values to differentiate the high- and low-expression groups.

### Statistical analysis

Non-normally distributed continuous variables were compared using the Mann–Whitney U test. The chi-square test or Fisher’s exact test was used to evaluate the relationship of neutrophil or macrophage infiltration or ETs with clinicopathological features and immune checkpoints, and a Spearman correlation analysis was for the non-normally distributed continuous variables. Kaplan–Meier plots were generated and compared using the log-rank test in GraphPad Prism software (v8.0.2, GraphPad Software, San Diego, CA, USA). Univariate and multivariate analyses were performed using a Cox proportional hazards regression model to calculate hazard ratios (HRs) with 95% confidence intervals (CI) for tumor progression and death. All statistical analyses were two-sided and were performed using SPSS (v22.0; IBM SPSS, Chicago, IL, USA), unless otherwise stated. Statistical significance was set at P < 0.05.

## Results

### The presence and characteristics of neutrophil and macrophage infiltration and ETs

**Figure 1:**
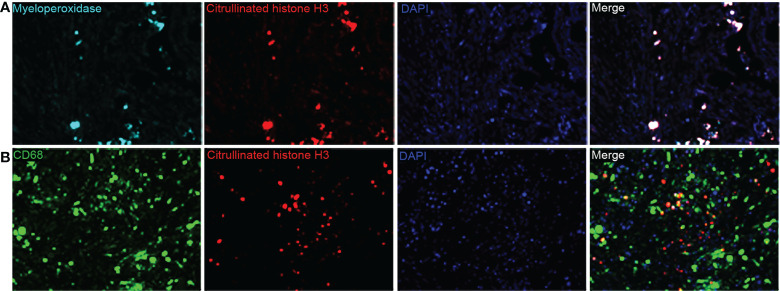
**(A)** Representative multiplex immunofluorescence staining images for myeloperoxidase-positive extracellular traps (ETs) and **(B)** macrophage ETs (magnification, 400×).

**Figure 2:**
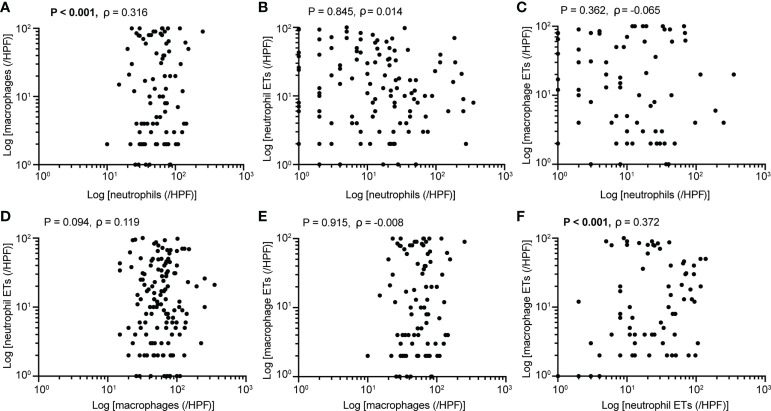
Associations between **(A)** neutrophil and macrophage infiltration (P<0.001), **(B)** neutrophil infiltration and ETs (P=0.845), **(C)** neutrophil infiltration and macrophage ETs (P=0.362), **(D)** macrophage infiltration and neutrophil ETs (P=0.094), **(E)** macrophage infiltration and ETs (P=0.915), **(F)** neutrophil and macrophage ETs (P<0.001) (Spearman’s correlation, n=205). P values <0.05 are bolded.

Baseline clinicopathological features of the PDAC cohort are shown in **Table 1**. We identified the presence of neutrophil and macrophage infiltration and ETs using immunochemistry and multiplex immunofluorescence staining (**Figure 1** and **Supplementary Figures S1** and **S2**). The densities of neutrophils and macrophages were as follows: neutrophils, median 10/HPF, interquartile range (IQR) 2.5–27.5/HPF; macrophage, median 54/HPF, IQR 34–71/HPF. The density of tumor-infiltrating macrophages was higher than that of tumor-infiltrating neutrophils (P < 0.001). Neutrophil infiltration was associated with moderate to good differentiation; infiltration of both neutrophils and macrophages was more frequently observed in tumors located in the head of the pancreas (**Supplementary Table S1**). We observed a significantly positive correlation between neutrophil and macrophage infiltration (**Figure 2A**).

**Table 1:** Associations between neutrophil or macrophage extracellular traps and clinicopathological features.

Variables	N	Neutrophil extracellular traps			N	Macrophage extracellular traps		
		Low	High	P value		Low	High	P value
Sex				0.778				0.798
Female	97	54 (56)	43 (44)		97	73 (75)	24 (26)	
Male	108	58 (54)	50 (46)		108	83 (77)	25 (23)	
Age, years				0.424				0.868
<60	90	52 (58)	38 (42)		90	69 (77)	21 (23)	
≧60	115	60 (52)	55 (48)		115	87 (76)	28 (24)	
Location				0.086				0.891
Head	128	64 (50)	64 (50)		128	97 (76)	31 (24)	
Body & neck	77	48 (62)	29 (38)		77	59 (77)	17 (231)	
Lymphovascular invasion				0.348				0.544
Absent	137	78 (57)	59 (43)		137	106 (77)	31 (23)	
Present	68	34 (50)	34 (50)		68	50 (73)	18 (27)	
Perineural invasion				0.478				0.915
Absent	74	38 (51)	36 (49		74	56 (76)	18 (24)	
Present	131	74 (57)	57 (43)		131	100(76)	31 (24)	
Tumor differentiation				0.337				0.257
Moderately/well-differentiated	135	77 (57)	58 (43)		135	106 (79)	29 (21)	
Poorly differentiated	70	35(50)	35 (50)		70	50 (71)	20 (29)	
Tumor stage				0.608				0.095
T1-2	153	82 (54)	71 (46)		153	112 (73)	41 (27)	
T3	52	30 (58)	22 (42)		52	44 (85)	8 (15)	
Lymph node metastasis				0.229				0.124
Absent	82	49 (60)	33 (40)		89	67 (82)	15 (18)	
Present	123	63 (51)	60 (49)		123	89 (72)	34 (28)	
Distant metastasis				0.817				0.673
M0	199	109 (55)	90 (45)		199	151 (76)	48 (24)	
M1	6	3 (50)	3 (50)		6	5 (83)	1 (17)	
AJCC stage				0.173				0.856
I-II	165	94 (57)	71 (43)		165	126 (76)	39 (24)	
III- IV	40	18 (45)	22 (55)		40	30 (75)	10 (25)	

AJCC, American Joint Committee on Cancer. AJCC stage was assessed according to the eighth edition of the American Joint Committee on Cancer guidelines.

The ET expression rates in macrophages and neutrophils were 23.9% and 45.4%, respectively. Positive staining for macrophage and neutrophil ETs was not related to clinicopathological characteristics, including age, sex, tumor differentiation, tumor stage, node stage, distant metastasis, or AJCC stage (**Table 1**). Positive staining for neutrophil ETs was not related to the infiltration of neutrophil infiltration, and also showed no association with the macrophage infiltration (**Figures 2B–F**). Positive staining for macrophage ETs was not related to infiltration of neutrophils or macrophages. Positive staining for macrophage ETs correlated with that for neutrophil ETs (**Figures 2B–F**).

### Effect of neutrophil and macrophage infiltration or ETs on survival rates

**Figure 3:**
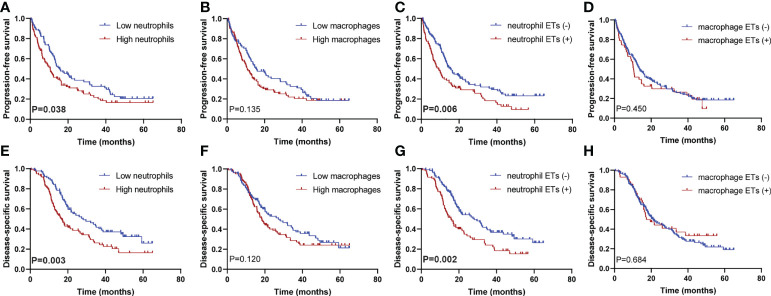
Kaplan–Meier survival curves according to neutrophil or macrophage infiltration or ETs. Progression-free survival for **(A)** neutrophil infiltration (P=0.038), **(B)** macrophage infiltration (P=0.135), **(C)** neutrophil ETs (P=0.006), and **(D)** macrophage ETs (P=0.450); disease-specific survival for **(E)** neutrophil infiltration (P=0.003), **(F)** macrophage infiltration (P=0.120), **(G)** neutrophil ETs (P=0.002), and **(H)** macrophage ETs (P=0.684) (Log-rank test, n=205). P values <0.05 are bolded.

The Kaplan–Meier curves showed that high densities of tumor-infiltrating neutrophils were associated with worse PFS and DSS than those at low densities; positive neutrophil ETs were also related to poorer PFS and DSS (**Figures 3A-H**). Neither macrophage infiltration nor ETs were correlated with PFS or DSS. Low differentiation grade, high T stage, high N stage, advanced AJCC stage, high count, and positive neutrophil ETs were associated with shorter PFS and DSS, whereas ACT was associated with improved PFS and DSS (**Table 2**). Low tumor-infiltrating neutrophil numbers and positive neutrophil ETs were predictors of improved DSS but not PFS, independent of AJCC stage, ACT, or tumor differentiation (**Table 3**).

**Table 2:** Univariate analysis of factors potentially predictive of survival in patients with pancreatic ductal adenocarcinoma.

Variables		Progression-free survival		Disease-specific survival	
		HR (95% CI)	P-value	HR (95% CI)	P-value
Age, years	< 60 vs ≥ 60	0.990 (0.711–1.379)	0.954	1.035 (0.727–1.474)	0.847
Tumor differentiation	Moderately/well-differentiated vs poorly differentiated	1.766 (1.260–2.476)	**0.001**	1.917 (1.341–2.739)	**<0.001**
Lymphovascular invasion	Absent vs present	1.174 (0.832–1.656)	0.363	1.088 (0.751–1.576)	0.657
Perineural invasion	Absent vs present	1.181 (0.834–1.672)	0.349	1.191 (0.822–1.726)	0.354
Tumor stage	T1-2 vs T3	1.689 (1.162–2.455)	**0.006**	1.756 (1.185–2.600)	**0.005**
Node stage	N0 vs N1-2	1.816 (1.277–2.582)	**0.001**	1.691 (1.161–2.461)	**0.006**
Distant metastasis	M0 vs M1	2.098 (0.856–2.141)	0.105	2.269 (0.922–5.580)	0.074
AJCC stage	I-II vs III-IV	2.088 (1.426–3.057)	**<0.001**	2.237 (1.508–3.318)	**<0.001**
Adjuvant chemotherapy	No vs yes	0.541 (0.382–0.768)	**0.001**	0.451 (0.214–0.650)	**<0.001**
Neutrophils, /HPF	Low vs high	1.433 (1.021–2.010)	**0.038**	1.723 (1.196–2.284)	**0.003**
Macrophages, /HPF	Low vs high	1.290 (0.923–1.804)	0.136	1.264 (0.883–1.808)	0.201
Neutrophil extracellular traps	Negative vs positive	1.576 (1.134–2.191)	**0.007**	1.758 (1.236–2.501)	**0.002**
Macrophage extracellular traps	Negative vs positive	1.160 (0.789–1.705)	0.451	0.914 (0.593–1.408)	0.914

AJCC, American Joint Committee on Cancer; CI, confidence interval; HR, hazard ratio.

P values <0.05 are bolded.

**Table 3:** Multivariate analysis of factors potentially predictive of survival in patients with pancreatic ductal adenocarcinoma.

Variables		Progression-free survival		Disease-specific survival	
		HR (95% CI)	P-value	HR (95% CI)	P-value
Tumor differentiation	Moderately/well-differentiated vs poorly differentiated	1.527 (1.061–2.196)	**0.023**	1.619 (1.107–2.368)	**0.013**
AJCC stage	I-II vs III-IV	1.756 (1.172–2.630)	**0.006**	1.907 (1.260–2.886)	**0.002**
Adjuvant chemotherapy	No vs yes	0.682 (0.464–1.002)	0.051	0.589 (0.394–0.883)	**0.010**
Neutrophils, /HPF	Low vs high	1.170 (0.822–1.606)	0.384	1.477 (1.017–2.145)	**0.041**
Neutrophil extracellular traps	Negative vs positive	1.353 (0.950–1.928)	0.094	1.487 (1.014–2.180)	**0.042**

AJCC, American Joint Committee on Cancer; CI, confidence interval; HR, hazard ratio.

P values <0.05 are bolded.

### Association of neutrophil infiltration and ETs with response to ACT

**Figure 4:**
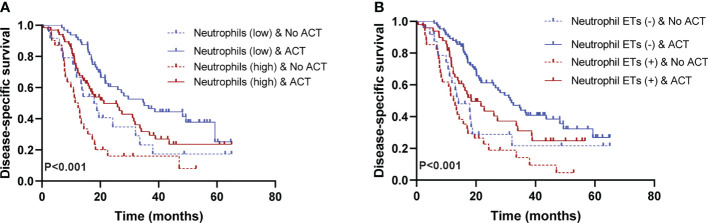
**(A)** Low neutrophil infiltration is associated with a positive response to adjuvant chemotherapy (ACT) (P<0.001). **(B)** Negative neutrophil ETs are associated with a positive response to ACT. Survival curves of ACT-treated patients with high/low neutrophil infiltration or positive/negative neutrophil ETs (P<0.001). (Log-rank test, n=205) ACT, adjuvant chemotherapy. P values <0.05 are bolded.

In this study, ACT was associated with favorable PFS and DSS (**Tables 2**, **3**), consistent with previous results (28). To assess whether patients with low/high neutrophil infiltration or negative/positive staining for neutrophil ETs respond differently to ACT, we tested the relationship between neutrophil infiltration or ETs and DSS among patients who did or did not receive ACT. Patients with low tumor-infiltrating neutrophil numbers or negative neutrophil ETs showed improved DSS after ACT (**Figures 4A, B**). A test for the interaction between the biomarker and the treatment demonstrated that the benefit observed in low neutrophil infiltration or negative staining for neutrophil ETs subgroups was superior to that observed in high neutrophil infiltration or positive staining for neutrophil ETs (**Table 4**).

**Table 4:** Results of the multivariate cox regression analysis of ACT per neutrophil infiltration and Neutrophil ETs subgroups.

ACT stratified by group	Subgroups	Disease-specific survival
Neutrophils		
	Low	HR 0.521, 95% CI 0.289–0.940, P=**0.030**
	High	HR 0.627, 95% CI 0.360–1.094, P=0.101
Neutrophil ETs		
	Negative	HR 0.413, 95% CI 0.234–0.730, P=**0.002**
	Positive	HR 0.785, 95% CI 0.445–1.386, P=0.404

ACT, adjuvant chemotherapy; ET, extracellular trap; HR, hazard ratio; CI, confidence interval.

P values <0.05 are bolded.

### Association between neutrophil infiltration and ETs and prognosis

We explored the association between clinical outcomes and the coexistence of high tumor-infiltrating neutrophil numbers and positive neutrophil ETs. We found that 57 (27.8%) patients had neutrophils (low)/neutrophil ETs (–), 52 (25.4%) had neutrophils (low)/neutrophil ETs (+), 37 (18.0%) had neutrophils (high)/neutrophil ETs (–), and 51 (24.9%) had neutrophils (high)/neutrophil ETs (+). Patients with neutrophils (high)/neutrophil ETs (+) showed the worst survival, whereas patients with neutrophils (low)/neutrophil ETs (–) showed the most favorable survival (P=0.019 for PFS, P<0.001 for DSS) (**Supplementary Figure S3A, B**).

### Neutrophil and macrophage infiltration and ETs across PD-L1-negative and -positive subgroups

The numbers of neutrophils in the PD-L1 TPS ≥ 1% subgroup were higher than those in the PD-L1 TPS < 1% subgroup. We did not observe any difference between the PD-L1 TPS ≥1% and PD-L1 TPS < 1% subgroups for macrophage ETs or infiltration of neutrophils and macrophages (**Supplementary Figures S4A–D**). The densities of macrophages were lower in the PD-L1 IC < 1% subgroup than in the PD-L1 IC ≥ 1% subgroup; there were no differences between the PD-L1 IC ≥ 1% and PD-L1 IC < 1% subgroups for neutrophil and macrophage ETs or neutrophil infiltration (**Supplementary Figures S4E–H**).

Patients in the PD-L1 TPS < 1% subgroup demonstrated lower DSS with positive neutrophil ETs than negative neutrophil ETs; no differences were observed in the DSS of the PD-L1 TPS < 1% subgroup for macrophage ETs or neutrophil and macrophage infiltration (**Supplementary Figures S5A–H**). High neutrophil infiltration was related to poor DSS in the PD-L1 TPS ≥ 1% subgroup (**Supplementary Figures S6A–H**). Patients in the PD-L1 IC < 1% subgroup demonstrated lower PFS and DSS with positive neutrophil ETs than negative neutrophil ETs; high neutrophil infiltration was related to unfavorable DSS in the PD-L1 IC < 1% subgroup; no difference in PFS or DSS was observed in the PD-L1 IC < 1% subgroup for macrophage ETs or infiltration (**Supplementary Figures S7A–H**); and no difference was observed in the PD-L1 IC ≥ 1% subgroup for neutrophil and macrophage infiltration or ETs (**Supplementary Figures S8A–H**).

### Association of neutrophil and macrophage infiltration and ETs with other immune checkpoints

We found that neutrophil ETs were associated with positive B7-H4 expression, and not with PD-L2 and B7-H3 expression (**Supplementary Tables 2**, **3**). The number of tumor-infiltrating neutrophils or macrophages was not related to the expression of PD-L2, B7-H3, or B7-H4; macrophage ETs showed similar results. Compared with the positive expression of B7-H3, the positive expression of PD-L1, PD-L2, and B7-H4 was associated with worse survival (29–31).

## Discussion

In the current study, we found that there were relatively fewer neutrophil and macrophage ETs than tumor-infiltrating neutrophils and macrophages in PDAC. Patients with PDAC were more accurately stratified based on the infiltration of neutrophils and presence of neutrophil ETs. Positive staining for neutrophil ETs and high density of tumor-infiltrating neutrophils were independent prognostic indicators of reduced DSS. Compared to macrophage ETs, neutrophil ETs were associated with the differential infiltration of immune cells. Neutrophil ETs were related to the infiltration of macrophages, but not neutrophils; macrophage ETs were not associated with neutrophil or macrophage infiltration. Neutrophil ETs were associated with positive B7-H4 expression. Patients in the PD-L1 TPS < 1% subgroup demonstrated lower DSS with positive neutrophil ETs than with negative neutrophil ETs. Furthermore, PDAC patients with low tumor-infiltrating neutrophil numbers or negative neutrophil ETs had higher DSS rates after ACT.

Neutrophil ETs have been investigated in several types of tumors, where they mainly serve as pro-tumor factors depending on the status of the tumor microenvironment and tumor heterogeneity (32). By multiplex immunofluorescence, we demonstrated that neutrophil ETs were associated with poor survival in patients with PDAC. Richardson et al. (33) revealed that the number of neutrophil ETs was higher in patients with colorectal cancer than in healthy controls and was related to an unfavorable prognosis. Xu et al. (12) found that neutrophil ETs were correlated with worst recurrence-free survival in non-functional pancreatic neuroendocrine tumors. Park et al. (34) showed that neutrophil ETs were observed more frequently in metastatic lung lesions with the highest expression in triple-negative tumors. Tohme et al. (35) found that neutrophil ETs were expressed in higher numbers in colorectal liver metastasis than in liver tissue. Based on these and our findings, we hypothesize that neutrophil ETs have an important influence on tumor progression. Previous studies have shown that tumor cell-induced neutrophil ETs could stimulate immune cells to release inflammatory cytokines, including IL-1, IL-6, ICAM-1, VCAM-1, and E-selectin, leading to tumor growth (36). Neutrophil ETs also contribute to growth, invasion, and metastasis by regulating mitochondrial function (37), and can promote T-cell exhaustion to foster a suppressive microenvironment (15). Thus, neutrophil ETs, through interaction with tumor cells and the immune microenvironment, promote tumor progression. Future studies should focus on the function and regulatory mechanisms of neutrophil ETs in PDAC.

To date, the role of macrophage ETs in tumors has rarely been explored. Xu et al. (12) found that the presence of macrophage ETs was related to reduced recurrence-free survival and was an independent risk factor in pancreatic neuroendocrine tumors, which varied from our results in PDAC. In the present study, macrophage ETs was not associated with prognosis. Positive staining for macrophage ETs was related to the presence of neutrophil ETs and was not related to clinicopathological parameters or the infiltration of neutrophils or macrophages. The molecular mechanisms of macrophage ET and its interaction with PDAC cells require further investigation.

We found that neutrophil density was a prognostic predictor of DSS and PFS, and, along with neutrophil ETs, tumor differentiation, AJCC stage, and ACT, also an independent factor for DSS. Consistent with our findings, Wang et al. (38) reported that the presence of CD15^+^ neutrophils was associated with shorter survival in a cohort of 79 patients with PDAC. Neutrophils can promote angiogenesis, suppress tumor-infiltrating T cells, and recruit macrophages and regulatory T cells, leading to tumor progression (39–41). This may partly explain the association of high neutrophil expression and poor prognosis in PDAC. Compared to neutrophil infiltration, the density of tumor-infiltrating macrophages was not correlated with survival, whereas Knudsen et al. (42) found that CD68^+^ macrophage levels were associated with poor prognosis. We propose that this inconsistency is related to differences in methodology, antibodies, and sample size or the fact that Knudsen et al. (42) did not detect neutrophil ETs and thus was not able to report a potential association between the infiltration of macrophages and the presence of neutrophil ETs. Our results showed that the density of macrophages was significantly associated with positive staining for neutrophil ETs; thus the interaction between macrophages and neutrophil ETs may synergistically contribute to PDAC tumor progression and the effect of macrophages on prognosis was potentially modified by neutrophil ETs.

Our findings also revealed the predictive significance of neutrophil infiltration or the presence of neutrophil ETs in ACT response. Patients with low neutrophil infiltration or negative staining for neutrophil ETs who received ACT had a higher DSS rate, which indicates that neutrophil infiltration or the presence of neutrophil ETs could be used as criteria to screen patient groups according to potential ACT response or serve as markers for more effective personalized treatments. However, further studies are needed to validate these results.

The effect of PD-1 blockade combined with DNase I to inhibit neutrophil ETs has gained increasing attention (16). In analyzing the association between neutrophil and macrophage ETs and immune checkpoint molecules, we found that patients in the PD-L1 TPS < 1% subgroup demonstrated lower DSS with positive neutrophil ETs than negative neutrophil ETs, and patients in the PD-L1 IC < 1% subgroup demonstrated lower PFS and DSS with positive neutrophil ETs than negative neutrophil ETs. Despite these controversial findings, PD-L1 expression levels have been used as a predictive biomarker to guide anti-PD-1 immunotherapy (43). The limited efficacy of anti-PD-1 therapy in PD-L1-negative tumors highlights the importance of identifying alternative or combination treatment strategies. Our results suggest that neutrophil ET may be a promising target for some PD-L1-negative patients, a subgroup resistant to anti-PD-1 therapy. Furthermore, neutrophil ETs were associated with B7-H4 expression. B7-H4 is a T-cell coinhibitory B7 family molecule and a potential therapeutic target in immunotherapies (44). Based on our data, the blockade of B7-H4 and inhibition of neutrophil ETs may provide an effective therapeutic option for PDAC. However, the regulatory mechanisms between neutrophil ETs and immune checkpoints require further in-depth investigation.

We acknowledge some limitations to our study. First, its retrospective nature has inherent limitations. Second, tumor heterogeneity is inevitable when using TMAs. Third, the small number of cases might be considered a further limitation of the subgroup analysis. Finally, this study was performed on patients from a single institution and lacked an independent validation cohort.

In conclusion, we found that a high number of neutrophils and positive staining for neutrophil ETs were associated with reduced survival and were independent predictors of DSS. Patients treated with ACT showed improved DSS in a population with low tumor-infiltrating neutrophil numbers or negative neutrophil ETs. Additionally, we found that four subgroups categorized by the infiltration of neutrophils and the presence of neutrophil ETs revealed distinct clinical outcomes. The number and prognostic value of neutrophil and macrophage infiltration or ETs varied between the PD-L1-negative and -positive subgroups. Furthermore, the presence of neutrophil ETs was related to B7-H4 expression. The role of neutrophil ETs in the clinical response to chemotherapy and immunotherapy in PDAC requires further investigation to improve the resistance to these therapy for patients with PDAC.

## Data availability statement

The data used in this study are available from the corresponding author upon reasonable request. Requests to access the datasets should be directed to JC, chenjie@pumch.cn.

## Ethics statement

This study was approved by the Institutional Review Board of the Peking Union Medical College Hospital (S-K1593) and was conducted in accordance with the Declaration of Helsinki.

## Author contributions

XC contributed to sample and data acquisition, and manuscript drafting. SM, HM, SY, and ZL provided technical support. JC made substantial contributions to the conception and design, funding, and supervision of the study. All authors contributed to the article and approved the submitted version.

## Funding

This work was supported by grants from the Chinese Academy of Medical Sciences Initiative for Innovative Medicine (CAMS-2016-I2M-1–001), National Natural Science Foundation of China (Nos. 81472326 and 81672648), and National Scientific Data Sharing Platform for Population and Health (NCMI-YF01N-201906).

## Conflict of interest

The authors declare that the research was conducted in the absence of any commercial or financial relationships that could be construed as a potential conflict of interest.

## Publisher’s note

All claims expressed in this article are solely those of the authors and do not necessarily represent those of their affiliated organizations, or those of the publisher, the editors and the reviewers. Any product that may be evaluated in this article, or claim that may be made by its manufacturer, is not guaranteed or endorsed by the publisher.
