# Supplementary material for: Intratumoral neutrophil extracellular traps are associated with unfavorable clinical outcomes and immunogenic context in pancreatic ductal adenocarcinoma
Source: Front Immunol. 2022 Oct 17;13:1027459. doi: 10.3389/fimmu.2022.1027459 (PMC9618733; doi:10.3389/fimmu.2022.1027459)

Supplementary Table S1. Associations between neutrophil or macrophage infiltration and clinicopathological features.

Variables	N	Neutrophil infiltration			N	Macrophage infiltration		
		Low	High	P value		Low	High	P value
Sex				0.540				0.115
Female	94	47 (50)	47 (50)		96	41 (43)	55 (57)	
Male	103	47 (46)	56 (54)		104	56 (54)	48 (46)	
Age, years				0.511				**0.041**
<60	87	39 (45)	48 (55)		89	36 (40)	53 (60)	
≧60	110	55 (50)	55 (50)		11	61 (55)	50 (45)	
Location				**0.049**				**0.026**
Head	125	53 (42)	72 (58)		127	54 (43)	73 (57)	
Body & neck	72	41 (57)	31 (43)		73	43 (59)	30 (41)	
Lymphovascular invasion				0.092				0.455
Absent	133	69 (52)	64 (48)		133	67 (50)	66 (50)	
Present	64	25 (39)	39 (61)		67	30 (45)	37 (55)	
Perineural invasion				0.095				0.750
Absent	72	40 (56)	32 (44)		72	36 (50)	36 (50)	
Present	125	54 (43)	71 (57)		128	61 (48)	67 (52)	
Tumor differentiation				**0.002**				0.365
Moderately/well-differentiated	134	74 (55)	60 (45)		134	68 (51)	66 (49)	
Poorly differentiated	63	20 (32)	43 (68)		66	29 (44)	37 (56)	
Tumor stage				0.264				0.462
T1-2	149	72 (48)	77 (52)		151	71 (47)	80 (53)	
T3	48	22 (46)	26 (54)		49	26 (53)	23 (47)	
Lymph node metastasis				0.603				**0.026**
Absent	78	39 (50)	39 (50)		79	46 (58)	33 (42)	
Present	119	55 (46)	64 (54)		121	51 (42)	70 (58)	
Distant metastasis				0.685				0.684
M0	191	92 (48)	99 (52)		194	95 (49)	99 (51)	
M1	6	2 (33)	4 (67)		6	2 (33)	4 (67)	
AJCC stage				0.459				0.120
I-II	157	77 (49)	80 (51)		160	82 (51)	78 (49)	
III- IV	40	17 (43)	23 (57)		40	15 (37)	25 (63)	

AJCC, American Joint Committee on Cancer. AJCC stage was assessed according to the eighth edition of the American Joint Committee on Cancer guidelines.
P values <0.05 are bolded.

Supplementary Table S2. Associations of neutrophil or macrophage infiltration with immune checkpoints.

Variables	N	Neutrophil infiltration			N	Macrophage infiltration		
		Low	High	P value		Low	High	P value
PD-L2				0.987				0.590
Negative	113	51 (45)	62 (55)		114	54 (47)	60 (53)	
Positive	60	27 (45)	33 (55)		60	31 (52)	29 (48)	
B7-H3				0.682				0.755
Negative	6	2 (33)	4 (67)		6	4 (67)	2 (33)	
Positive	165	82 (50)	83 (50)		163	71 (44)	92 (56)	
B7-H4				0.352				0.191
Negative	49	25 (51)	24 (49)		50	28(56)	22 (44)	
Positive	134	58 (43)	76 (57)		135	61 (45)	74 (55)	

B7-H3, B7 homolog 3; B7-H4, B7 homolog 4; PD-L2, programmed cell death-ligand 2.

Supplementary Table S3. Associations of neutrophil or macrophage extracellular traps with immune checkpoints.

Variables	N	Neutrophil extracellular traps			N	Macrophage extracellular traps		
		Low	High	P value		Low	High	P value
PD-L2				0.660				0.851
Negative	114	61 (54)	53 (46)		114	84 (74)	30 (26)	
Positive	60	30 (50)	30 (50)		60	45 (75)	15 (25)	
B7-H3				0.501				0.155
Negative	6	4 (67)	2 (33)		6	6 (100)	0 (0)	
Positive	165	87 (53)	78 (47)		165	123 (75)	42 (25)	
B7-H4				**0.017**				0.352
Negative	50	35 (70)	15 (30)		50	40 (80)	10 (20)	
Positive	135	68 (50)	67 (50)		135	99 (73)	36 (27)	

B7-H3, B7 homolog 3; B7-H4, B7 homolog 4; PD-L2, programmed cell death-ligand 2.
P values <0.05 are bolded.

Supplementary Figure S1. Representative immunohistochemical staining for neutrophils (CD15). (B) Representative immunohistochemical staining for macrophages (CD68). (magnification, 400×)

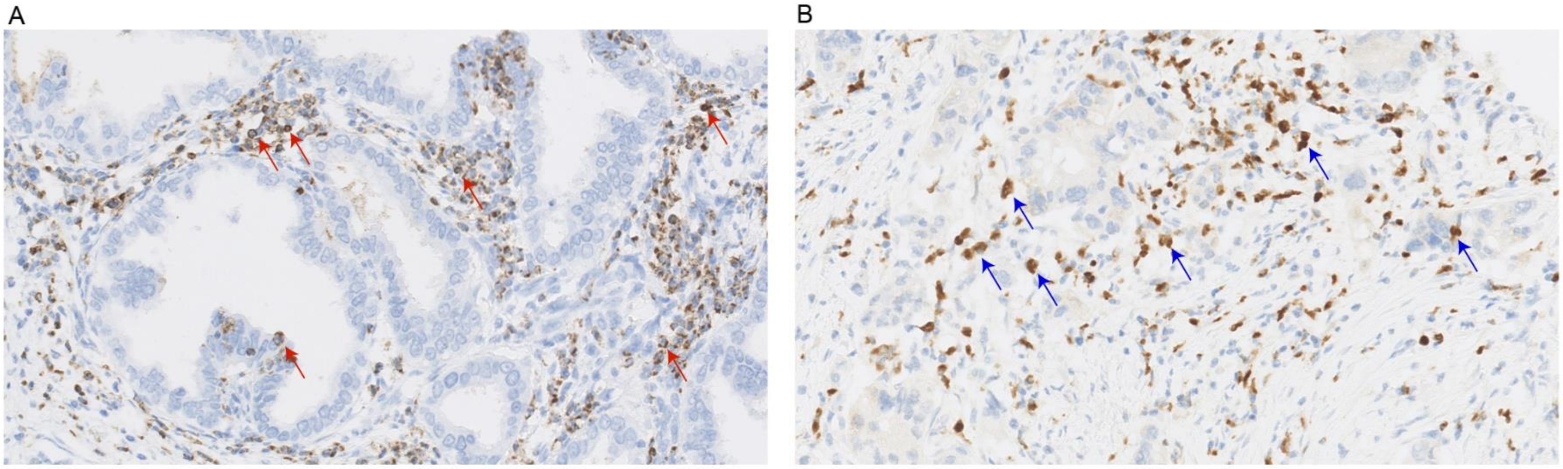

Supplementary Figure S2. ET staining in the same field illustrating the concomitant presence of neutrophil ETs and macrophages ETs. (A)Samples with intermediate values for both neutrophil ETs and macrophages ETs are present. (B) Samples with high content of neutrophil ETs have not high macrophage ETs content. ET, extracellular traps; MPO, myeloperoxidase; H3Cit, citrullinated histone H3.

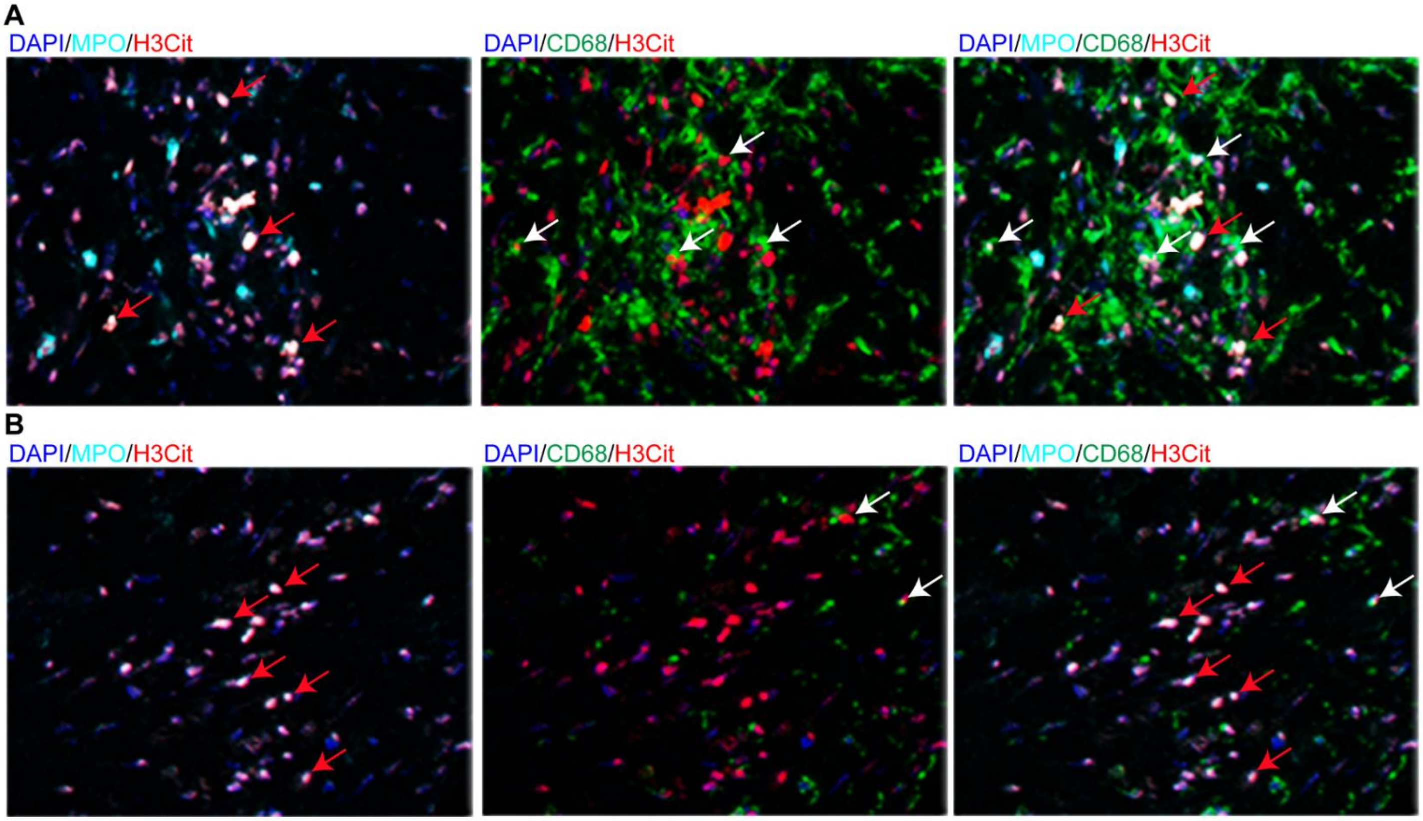

Supplementary Figure S3. Kaplan–Meier curves of PFS (A) (P=0.019) and DSS (B) (P=0.001) in patients based on the combination of neutrophil infiltration and neutrophil extracellular traps. (Log-rank test) P values <0.05 are bolded.

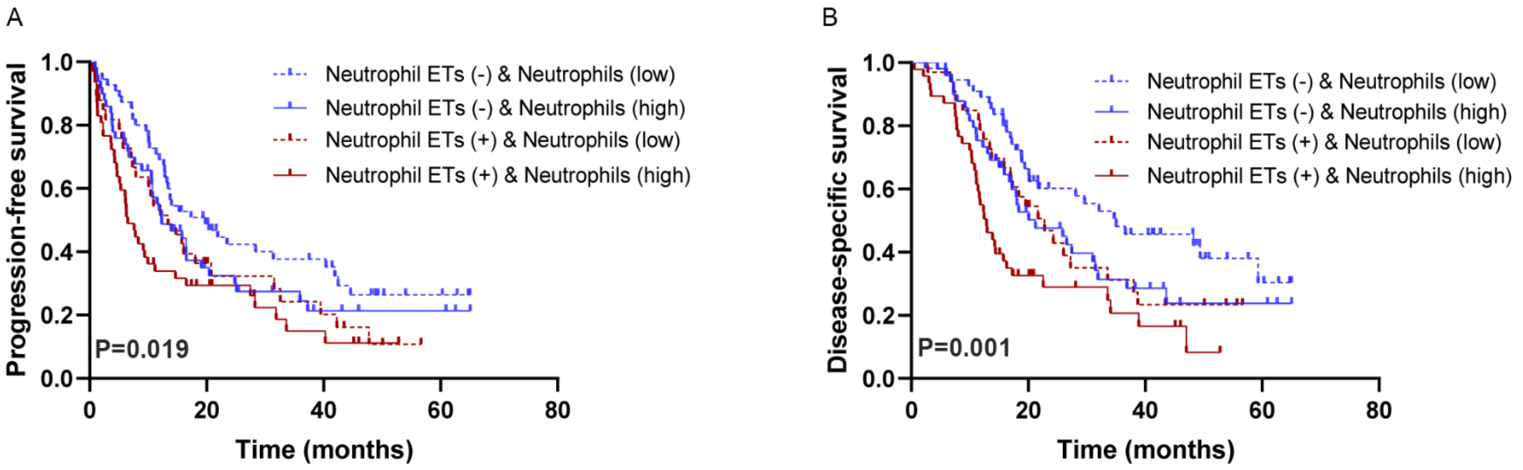

Supplementary Figure S4. (A) Comparison of neutrophil infiltration between PD-L1 TPS<1% and PD-L1 TPS⩾1% subgroups (P=0.478); (B) comparison of macrophage infiltration between PD-L1 TPS<1% and PD-L1 TPS⩾1% subgroups (P=0.062); (C) comparison of neutrophil extracellular traps between PD-L1 TPS<1% and PD-L1 TPS⩾1% subgroups (P=0.001); (D) comparison of macrophage extracellular traps between PD-L1 TPS<1% and PD-L1 TPS⩾1% subgroups (P=0.251); (E) comparison of neutrophil infiltration between PD-L1 IC<1% and PD-L1 IC⩾1% subgroups (P=0.761); (F) comparison of macrophage infiltration between PD-L1 IC<1% and PD-L1 IC⩾1% subgroups (P=0.008); (G) comparison of neutrophil extracellular traps between PD-L1 IC<1% and PD-L1 IC⩾1% subgroups (P=0.107); (H) comparison of macrophage extracellular traps between PD-L1 IC<1% and PD-L1 IC⩾1% subgroups (P=0.199). (Mann Whitney test) P values <0.05 are bolded.

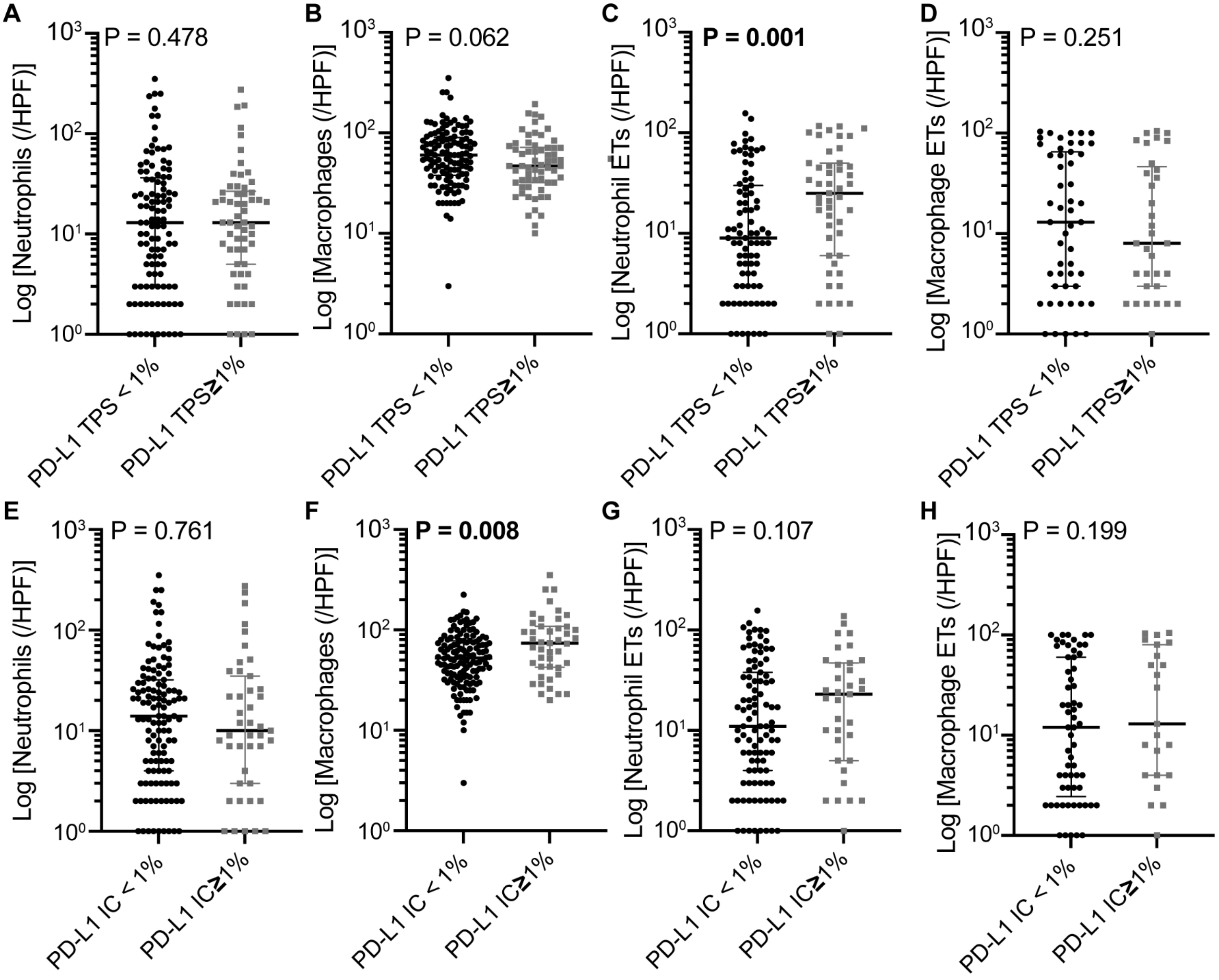

Supplementary Figure S5. Kaplan–Meier curves according to neutrophil or macrophage infiltration or extracellular traps in PD-L1 TPS<1% subgroup. Progression-free survival for (A) neutrophil infiltration (P=0.248), (B) macrophage infiltration (P=0.253), (C) neutrophil extracellular traps (P=0.068), and (D) macrophage extracellular traps in PD-L1 TPS<1% subgroup (P=0.405); disease-specific survival for (E) neutrophil infiltration (P=0.110), (F) macrophage infiltration (P=0.256), (G) neutrophil extracellular traps (P=0.037), and (H) macrophage extracellular traps in PD-L1 TPS<1% subgroup (P=0.692). (Log-rank test) P values <0.05 are bolded.

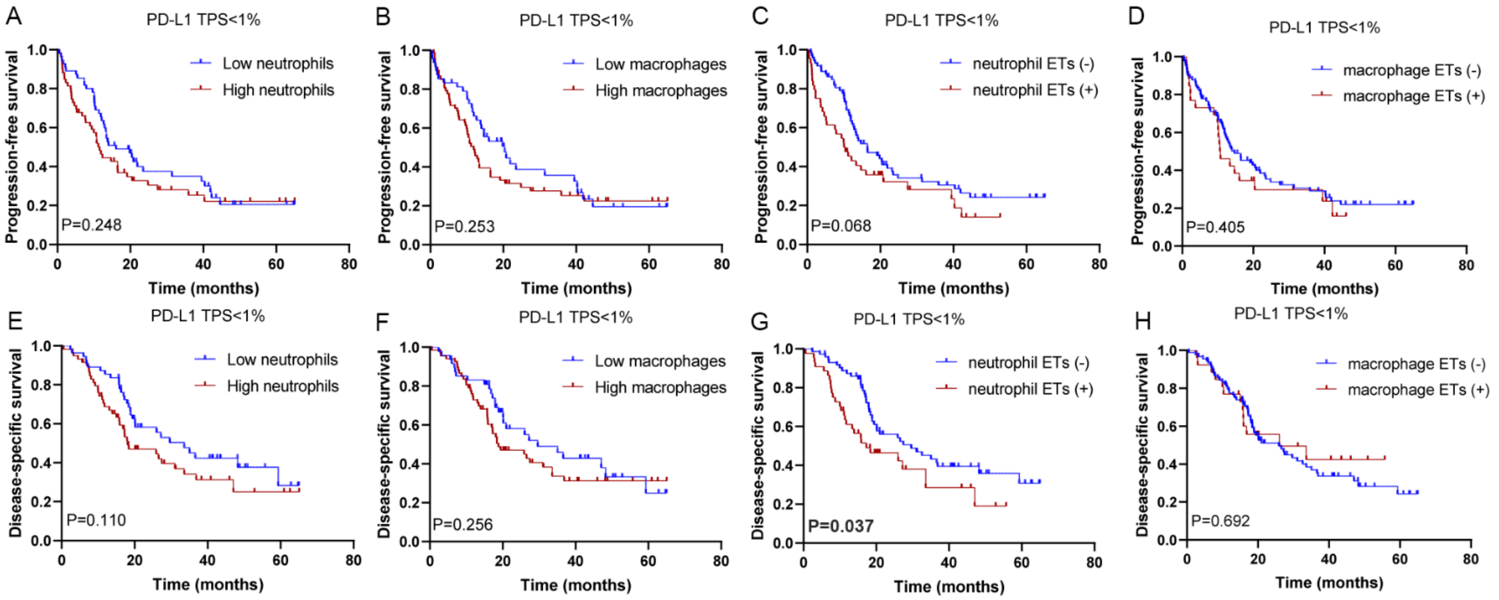

Supplementary Figure S6. Kaplan–Meier curves according to neutrophil or macrophage infiltration or extracellular traps in PD-L1 TPS ⩾1% subgroup. Progression-free survival for (A) neutrophil infiltration (P=0.104), (B) macrophage infiltration (P=0.089), (C) neutrophil extracellular traps (P=0.442), and (D) macrophage extracellular traps in PD-L1 TPS⩾1% subgroup (P=0.830); disease-specific survival for (E) neutrophil infiltration (P=0.041), (F) macrophage infiltration (P=0.131), (G) neutrophil extracellular traps (P=0.538), and (H) macrophage extracellular traps in PD-L1 TPS⩾1% subgroup (P=0.718). (Log-rank test) P values <0.05 are bolded.

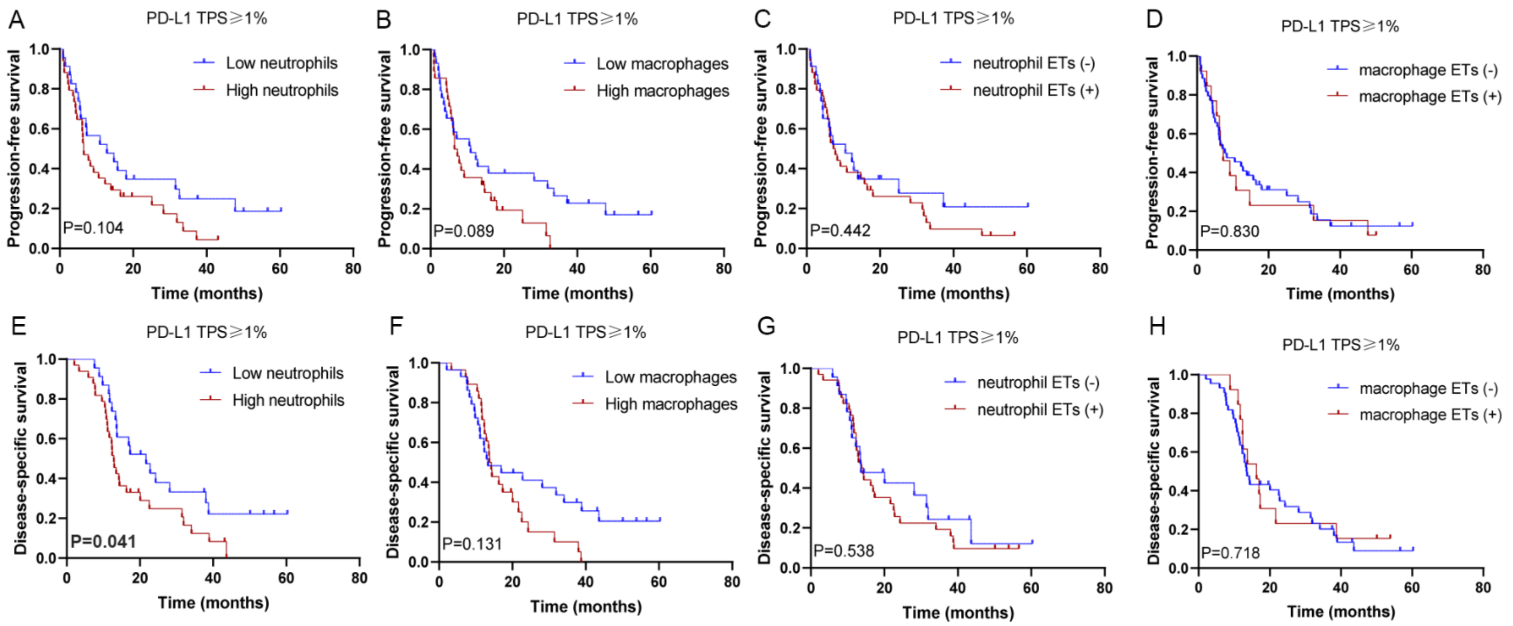

Supplementary Figure S7. Kaplan–Meier curves according to neutrophil or macrophage infiltration or extracellular traps in PD-L1 IC<1% subgroup. Progression-free survival for (A) neutrophil infiltration (P=0.057), (B) macrophage infiltration (P=0.110), (C) neutrophil extracellular traps (P=0.037), and (D) macrophage extracellular traps in PD-L1 IC<1% subgroup (P=0.246); disease-specific survival for (E) neutrophil infiltration (P=0.008), (F) macrophage infiltration (P=0.161), (G) neutrophil extracellular traps (P=0.012), and (H) macrophage extracellular traps in PD-L1 IC<1% subgroup (P=0.777). (Log-rank test) P values <0.05 are bolded.

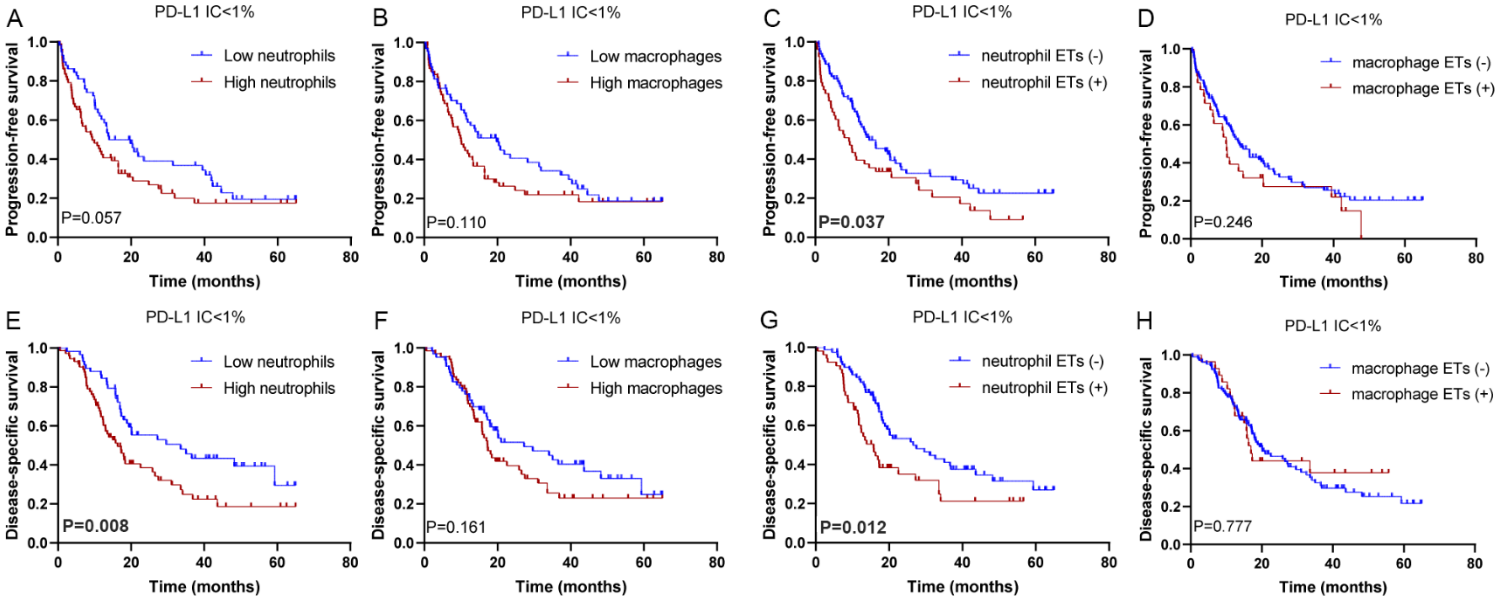

Supplementary Figure S8. Kaplan–Meier curves according to neutrophil or macrophage infiltration or extracellular traps in PD-L1 IC ⩾1% subgroup. Progression-free survival for (A) neutrophil infiltration (P=0.574), (B) macrophage infiltration (P=0.704), (C) neutrophil extracellular traps (P=0.297), and (D) macrophage extracellular traps in PD-L1 IC⩾1% subgroup (P=0.897); disease-specific survival for (E) neutrophil infiltration (P=0.550), (F) macrophage infiltration (P=0.655), (G) neutrophil extracellular traps (P=0.285), and (H) macrophage extracellular traps in PD-L1 IC⩾1% subgroup (P=0.731). (Log-rank test)

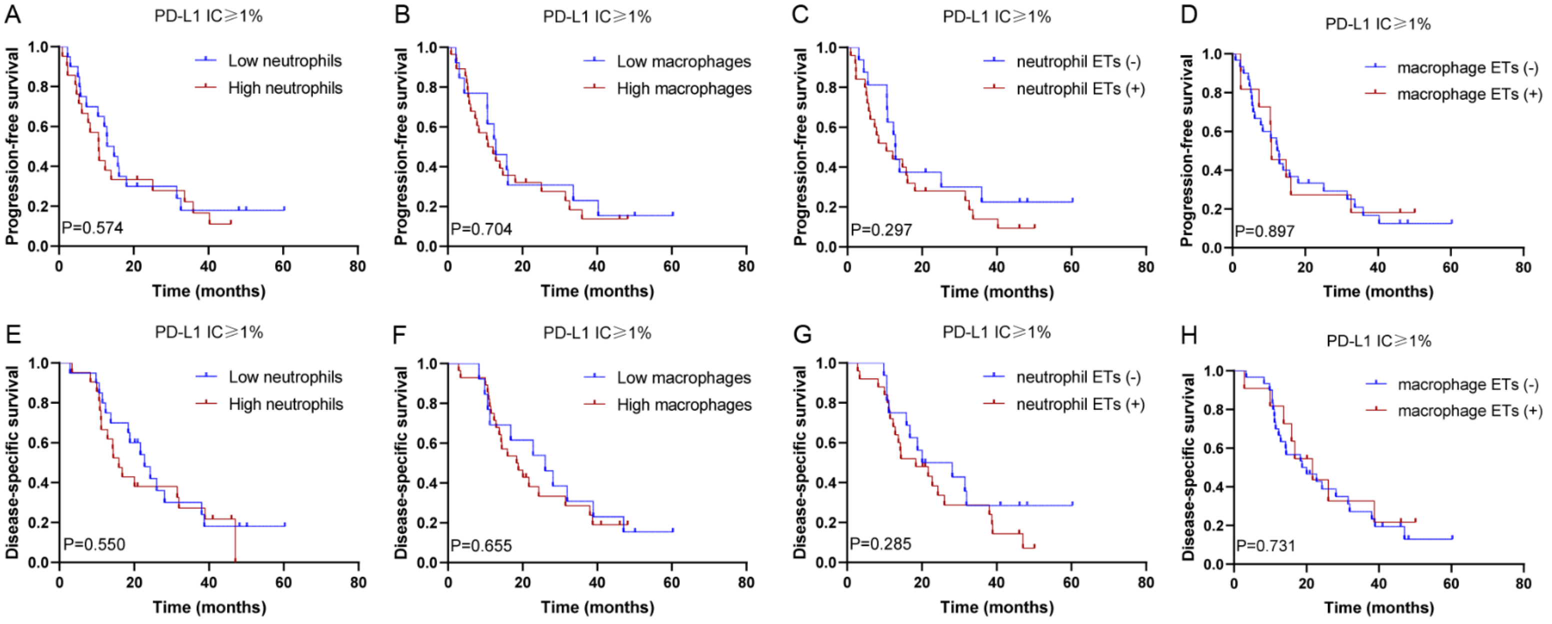

Supplement: Supplementary file 1 [file DataSheet_1.pdf]
